# Relationship of activities outside work to sleep and depression/anxiety disorders in Korean workers: the 4th Korean working condition survey

**DOI:** 10.1186/s40557-017-0206-8

**Published:** 2017-10-11

**Authors:** Sung Won Jung, Kyung-Jae Lee, Hee Sung Lee, Guang Hwi Kim, Jae Gwang Lee, June-Hee Lee, Joo Ja Kim

**Affiliations:** Department of Occupational & Environmental Medicine, Soonchunhyang University Hospital, Seoul, South Korea

**Keywords:** Activities outside work, Leisure activity, Social activity, Depression, Anxiety, Sleep disorder

## Abstract

**Background:**

Sleep disorders and depression/anxiety disorders are long-standing and significant problem for mental health. Also there are already known so many negative health effect of these disorders. But there were few studies to examine the association between activities outside work and forementioned disorders. So this study aimed the association of those things by using the Republic of Korean data.

**Methods:**

Data from 32,232 wage workers were used in the 4th Korean Working Condition Survey. General and occupational characteristics, sleep disorders, depression/anxiety disorders and activities outside work are included in questionnaire. To find the relationship between activities outside work and sleep, depression/anxiety disorders, multivariate logistic regression analysis was used after adjusting for general and occupational characteristics.

**Results:**

We observed that volunteer activities increased the odds ratio of both sleep disorders and depression/anxiety disorders(Odds ratio[OR] = 1.35, 95% confidence interval[CI]: 1.03–1.78 and OR = 1.54, 95% CI: 1.29–1.84, respectively). And self-development activities increase the odds ratio of sleep disorders(OR = 1.35, 95% CI: 1.17–1.57). Gardening activities lowered the odds ratio of depression/anxiety disorders(OR = 0.74, 95% CI: 0.59–0.94).

**Conclusion:**

Some of activities outside work were related to sleep disorders and depression/anxiety disorders among Korean wage workers. Our results showed negative health effect of some kinds of activities outside work such as volunteering and self-devlopment compared to other studies that emphasized positive effect of those activities for health.

## Background

Mental disorders are widely recognized as a significant issue in modern society, and according to a recent study, approximately 17% of South Korean citizens have experienced either a mental disorder or illness [1]. Figures from the Korea Informative Classification of Diseases (KOICD) report that over 2,000,000 patients were either hospitalized or clinically treated for mental disorders between 2008 and 2014, with a steadily increasing number of patients each year. The percentage of sleep disorders and depression/anxiety disorders is high among mental disorders, covering close to half of all mental illnesses (http://www.koicd.kr/stat/diseaseStats.do).

A large body of research studying the various effects of sleep disorders and depression/anxiety disorders has been produced over the years. Results show that sleep disorder is an illness that lowers work efficiency and quality of life [2]. A sleep disorder can also be a risk factor contributing to obesity and cardiovascular disease, among other various diseases [3, 4]. Furthermore, sleep disorders are one of the main causes of traffic accidents and can lead to an increase in suicides [5, 6]. This will undoubtedly become a considerable socioeconomic burden to bear [7].

The above is also evident in depression/anxiety disorders. They can become risk factors to various life threatening diseases, such as cardiovascular disease, osteoporosis, and heart failure [8–10]. If the degree of depression and anxiety during pregnancy is high, it may also cause perinatal problems such as premature birth, neonatal death, and maternal complications [11]. Furthermore, the risk of suicide increases if an individual is suffering from a depression/anxiety disorder [12].

Therefore, not only is this a significant public health problem, but it can also have a profound socioeconomic impact [13]. If such disorders are ignored, without adequate care, it will lead to chronic suffering and lower the quality of life. Thus, the need to emphasize prevention and treatment of depression/anxiety disorders is more important now than ever [14].

The aforementioned issues have led to many studies focused on identifying risk factors of sleep and depression/anxiety disorders. Among such studies, the bulk of the research is aimed towards identifying correlations between occupational factors [2, 15–17] and general sociological factors [18–20]. However, there is a shortage of research examining the correlation of non-occupational factors such as activities outside work and the aforementioned disorders, especially research utilizing data from the Republic of Korea.

Activities outside work, such as leisure activity can be defined as social and physical activities that require little effort [21]. Enjoying some free time after work or leisure activities on weekends can be a great opportunity to recover and regain energy [22]. Examples of common activities outside work from previous study results include volunteering, education, training, self-development, gardening, cultural activities, and sports [23]. The ‘Fourth Korean Working Conditions Survey (KWCS)’ questionnaire included questions related to the list of activities outside work previously mentioned.

There are various health benefits to be gained from engaging in activities outside work, and previous studies show that such activities have a positive effect on mental disorders. The aim of this study is to investigate the effects of activities outside work on sleep and depression/anxiety disorders in employed workers.

## Methods

### Study subjects

This research used data from the ‘Fourth Korean Working Conditions Survey (KWCS)’ conducted by the Occupational Safety and Health Research Institute (OSHRI) in 2014. This survey was developed based on the ‘European Working Conditions Survey’ conducted across Europe in 2010. The survey performed door-to-door interviews targeting wage workers aged 15 and over; as a result, 50,007 people were surveyed for the KWCS. Respondents that provided insufficient answers, such as ‘do not know’, or those who either did not or refused to respond were excluded. As wage workers were the target population, self-employed workers, employers, unpaid family/household members, and other workers were excluded. It also excluded the low number of respondents in the occupational category that are serving in the military or employed as agriculture and forestry workers, and respondents in the ‘between 15 and 19’ age group were excluded. In total, 32,232 wage workers over the age 20 or above became the sample of the survey.

### Measurements

#### General and occupational characteristics

General characteristics included gender, age (20–29 years, 30–39 years, 40–49 years, 50–59 years, or ≥60 years), and level of education (did not complete middle school, high school graduate, and university graduate and above). Occupational characteristics included employment status (regular work or temporary work/day labor), type of occupation (management/professional, regular office work, service/sales, technical, or manual/simple labor), weekly work hours (≤40, 41–48, 49–60, or >60), shift work flexibility, size of workplace (<50, 50–299, or ≥300), monthly income (<1,300,000 Korean won [KRW],1,300,000–1,990,000 KRW, 2,000,000–2,990,000 KRW, or ≥3,000,000 KRW). It was also assessed whether there was sufficient time to engage in activities outside of work hours.

#### Activities outside work

Activities outside work were independent variables. Individuals responded to the question of “In general, how often are you involved in any of the following activities outside work?” Activities outisde work include volunteer work, self-developtmnet, sports, outdoor/cultural activities or gardening. Answers including "more than an hour every day," "less than an hour every day or two," “once or twice a week,” “once or twice a month,” or “once or twice a year” were considered to have “taken part” in a activity outside work [14].

#### Sleep disorder and depression/anxiety disorders

Sleep disorders and depression/anxiety disorders, as dependable variables, were deemed to be apparent when a person answers ‘yes’ to the sub-items “Insomnia or sleep disorder” and “Depression or anxiety” from the question "Have you had any of the following problems during the past 12 months?".

#### Data analysis

First, a chi-square test was utilized to determine the distribution of employed workers with sleep disorders and depression/anxiety disorders according to their general and occupational characteristics. Next, chi-square test was used to determine the distribution of sleep disorders and depression/anxiety disorders according to activities outside work.

Next, the study attempted to analyze the relationship between activities outside work as independent variables with sleep disorders and depression/anxiety disorders as dependent variables. Multivariate logistic regression analysis was performed by adjusting general characteristics and occupational characteristics. The significance level was 0.05. All statistical analyses were performed using the SPSS software (version 14.0; SPSS Inc., Chicago, IL).

## Results

### Distribution of sleep disorders and depression/anxiety disorders according to general and occupational characteristics

Women had a higher percentage of sleep disorders (3.1%) and depression/anxiety disorders (1.6%) than men did. As age increased, there were higher chances of sleep disorders and depression/anxiety disorders. Lower levels of education also showed high chances of sleep disorders and depression/anxiety disorders. Regarding types of occupation, manual/simple labor had a higher proportion of sleep disorders (3.4%) and depression/anxiety disorders (1.9%) than other subtypes of workers. In terms of employment status, the proportion of non-regular workers was significantly higher than that of regular workers (1.6%) in depression/anxiety disorders, but about sleep disorders, there were no significant difference between the two groups. The longer the weekly working hours, the higher the percentage of sleep disorders (3.9%), but depression/anxiety disorders did not show any significant difference. In terms of size of workplace, the sleep disorder rate was the highest (3.5%) in medium sized business (50–299 employees). The sleep disorder rate was higher (4.7%) in shift work. The lower the income level, the higher the proportion of depression/anxiety disorders (1.8%), while there were no significant differences regarding sleep disorders and income level. Sleep disorders (4.8%) and depression/anxiety disorders (2.1%) were significantly higher in those who did not have enough time to do other activities outside of working hours. (Table 1).

**Table 1:** Number of workers with sleep disorders and depressive/anxiety disorders by general and occupational characteristics

			Depression/Anxiety disorder					Sleep disorder				
		Total (*N* = 32,232)	Yes		No		*P*- value^a^	Yes		No		*P*- value^a^
			*N*	(%)	*N*	(%)		*N*	(%)	*N*	(%)	
Gender												
Male		16,513	154	0.9	16,359	99.1	<0.001^†^	437	2.6	16,075	97.4	0.02^†^
Female		15,719	253	1.6	15,467	98.4		484	3.1	15,236	96.9	
Age												
20–29		4233	36	0.9	4197	99.1	<0.001^†^	113	2.7	4119	97.3	<0.001^†^
30–39		8463	92	1.1	8371	98.9		183	2.2	8280	97.8	
40–49		9406	119	1.3	9287	98.7		307	3.3	9099	96.7	
50–59		6456	85	1.3	6371	98.7		172	2.8	6274	97.2	
≥ 60		3674	75	2.0	3599	98.0		136	3.7	3538	96.3	
Education												
Middle school or below		3654	70	1.9	3584	98.1	<0.001^†^	123	3.4	3530	96.6	0.03^†^
High school		11,753	155	1.3	11,598	98.7		351	3.0	11,402	97.0	
University or above		16,825	181	1.1	16,644	98.9		447	2.7	16,379	97.3	
Occupation type												
Management/professional		3457	36	0.1	3421	99	<0.001^†^	92	2.6	3365	97.4	0.039^†^
Office work		9198	104	1.1	9094	98.9		242	2.6	8957	97.4	
Technical		6797	67	1.0	6730	99.0		178	2.6	6619	97.4	
Service/sales		7873	109	1.4	7764	98.6		245	3.1	7628	96.9	
Simple labor		4907	91	1.9	4816	98.1		165	3.4	4741	96.6	
Employment status												
Regular work		24,509	284	1.2	24,225	98.8	0.003^†^	708	2.9	23,801	97.1	0.601
Temporary worker		7723	123	1.6	7600	98.4		214	2.8	7509	97.2	
Working hours (/week)												
< 40		17,040	191	1.1	16,849	98.9	0.065	403	2.4	16,638	97.6	<0.001^†^
40–59		9406	131	1.4	9275	98.6		296	3.1	9110	96.9	
≥ 60		5786	84	1.5	5702	98.5		223	3.9	5562	96.1	
Number of employees												
< 50		23,486	297	1.3	23,189	98.7	0.848	626	2.7	22,860	97.3	0.002^†^
50–299		6168	81	1.3	6087	98.7		216	3.5	5952	96.5	
≥ 300		2578	30	1.2	2548	98.8		80	3.1	2498	96.9	
Shift work												
No		29,049	360	1.2	28,689	98.8	0.322	771	2.7	28,279	97.3	<0.001^†^
Yes		3183	47	1.4	3136	98.6		151	4.7	3031	95.3	
Income(10,000/month)												
< 130		6809	120	1.8	6689	98.2	<0.001^†^	218	3.2	6592	96.8	0.078
130–199		8211	120	1.5	8091	98.5		237	2.9	7974	97.1	
200–299		9308	86	0.9	9222	99.1		235	2.5	9073	97.5	
≥ 300		7904	81	1.0	7823	99.0		232	2.9	7671	97.1	
Enough time after work												
No		7885	166	2.1	7719	97.9	<0.001^†^	380	4.8	7505	95.2	<0.001^†^
Yes		24,347	241	1.0	24,106	99.0		542	2.2	23,805	97.8	

^a^Calculated using chi-square test

^†^ *P* < 0.05

### Distribution of sleep disorders and depression/anxiety disorders according to activities outside work

A chi-square test was performed to determine the distribution of sleep disorders and depression/anxiety disorders according to activities outside work. The proportion of sleep disorders were significantly higher (4.0%) in those that performed volunteering than in those that did not volunteer, but there was no significant difference between the two groups in depression/anxiety disorders.

Similarly, the proportion of sleep disorders were significantly higher (3.2%) in those that did self-development activities than those that did not partake in self-development activities, and there was no difference between the two groups in depression/anxiety disorders.

The proportion of depression/anxiety disorders was significantly lower (1.0%) in those who participated in gardening activities than in those who did not, while the difference in sleep disorders was not significant between the two groups. (Table 2).

**Table 2:** Distribution of sleep disorders and depression/anxiety disorders by non-occupational behavior

		Depression/anxiety disorder					Sleep disorder				
Total (*n* = 32,232)		Yes		No		*P*-value^a^	Yes		No		*P*-value^a^
		*N*	(%)	*N*	(%)		*N*	(%)	*N*	(%)	
Volunteering											
No	27,828	341	(1.2)	27,487	(98.8)	0.126	747	(2.7)	27,080	(97.3)	<0.001^†^
Yes	4404	66	(1.5)	4338	(98.5)		174	(4.0)	4231	(96.0)	
Self-development											
No	20,529	270	(1.3)	20,259	(98.7)	0.236	547	(2.7)	19,982	(97.3)	0.006^†^
Yes	11,703	136	(1.2)	11,566	(98.8)		374	(3.2)	11,329	(96.8)	
Leisure time activity											
No	10,382	162	(1.6)	10,220	(98.4)	0.001^†^	289	(2.8)	10,092	(97.2)	0.585
Yes	21,850	244	(1.1)	21,606	(98.9)		632	(2.9)	21,218	(97.1)	
Gardening											
No	23,015	313	(1.4)	22,707	(98.6)	0.013^†^	667	(2.9)	22,348	(97.1)	0.488
Yes	9217	94	(1.0)	9123	(99.0)		254	(2.8)	8963	(97.2)	

^a^Calculated using chi-square test

^†^ *P* < 0.05

### Relationship between activities outside work and depression/anxiety disorders

Multivariate logistic regression analysis was performed to determine the relationship between depression/anxiety disorders according to activities outside work. The previously mentioned general and occupational characteristics were adjusted and analyzed. As a result, it was found that the odds ratio of depression/anxiety disorder was significantly higher in individuals who volunteered compared to non-volunteers (OR = 1.35 [95% CI: 1.03–1.78]). In the case of gardening activities, it was found that the odds ratio of depression/anxiety disorders were significantly lower in those that did partake than in those that did not (OR = 0.74 [95% CI: 0.59–0.94]).(Table 3).

**Table 3:** Odds ratios of variables associated with depression/anxiety disorders

	Depression/anxiety disorder(N)		Unadjusted		Adjusted^a^	
	Yes	No	OR	95% CI	OR	95% CI
Volunteering	66	4338	1.23	0.94–1.60	1.35	1.03–1.78
(Ref: No)	341	27,487				
Self-development	136	11,566	0.89	0.71–1.09	1.07	0.85–1.35
(Ref: No)	244	20,259				
Leisure time activity	244	21,606	0.71	0.58–0.87	0.82	0.66–1.02
(Ref: No)	162	10,220				
Gardening	94	9123	0.74	0.59–0.93	0.74	0.59–0.94
(Ref: No)	313	22,707				

Calculated using multiple logistic regression analysis

^a^Adjusted for age, sex, education, employment status, number of employees, working hours, shift work, monthly income, and enough time after work

*OR* odds ratio, *CI* confidence interval

### The relationship between activities outside work and sleep disorders

Multivariate logistic regression analysis was performed to determine the relationship of sleep disorders with activities outside work. The previously mentioned general and occupational characteristics were adjusted.

It was confirmed that the odds ratio of sleep disorders was significantly higher in those that did partake in volunteering and self-development activities than in those that did not (respectively: OR = 1.54 [95% CI: 1.29–1.84]; OR = 1.35 [95% CI: 1.17–1.57]). (Table 4).

**Table 4:** Odds ratios of variables associated with sleep disorders

	Sleep disorders(N)		Unadjusted		Adjusted^a^	
	Yes	No	OR	95% CI	OR	95% CI
Volunteering	174	4231	1.49	1.26–1.76	1.54	1.29–1.84
(Ref: No)	747	27,080				
Self-development	374	11,329	1.21	1.05–1.34	1.35	1.17–1.57
(Ref: No)	547	19,982				
Leisure time activity	632	21,218	1.04	0.90–1.20	1.12	0.96–1.29
(Ref: No)	289	10,092				
Gardening	254	8963	0.95	0.82–1.10	0.94	0.81–1.09
(Ref: No)	667	22,348				

Calculated using multiple logistic regression analysis

^a^Adjusted for age, sex, education, employment status, number of employees, working hours, shift work, monthly income, and enough time after work

*OR* odds ratio, *CI* confidence interval

## Discussion

The study found what relationship activities outside work, such as volunteering, self-development activities, sports/cultural activities, and gardening activities, had with the mental disorders of Korean wage workers, especially sleep disorders and depression/anxiety disorders.

In this study, volunteer activities increased the odds ratio of sleep disorders and depression/anxiety disorders. However, previous studies reported that participation in volunteer activities increased sleep quality and contributed to health improvements [23]. In addition, some studies reported that volunteer activities, especially in the elderly, helped to alleviate and prevent depression/anxiety disorders. Other studies claimed that volunteering by young adults aged between 18 and 42 reported less depressive symptoms [24]. There are many studies on the benefits of volunteering; however, this study was one of the few that ended up showing negative health effects.

The contrary results to previous studies in our research could be due to reasons for volunteering, as volunteering participation in South Korea is not motivated by pure voluntary motives but rather by involuntary motives. To elaborate, the motivation for volunteering in numerous cases in South Korea is not to seek or achieve some sense of accomplishment or satisfaction, but is driven by a type of social motivation, such as a tool for employment or promotion. For employers, corporate social responsibility (CSR) may be factor. CSR is the responsibility of the company towards the community and the environment [25]. It has been found that CSR can have a negative effect on employees as companies force employee participation in volunteering activities.

In this study, self-development activities increase the odds ratio of sleep disorders. In previous studies, it was reported that the lower mental effort in the evening brought bettered the subjective quality of sleep and improvements in health [22]. In the case of Korean wage workers, it is thought that the above results were present because many self-development activities, such as education and training, take place in the evening after work [26].

This study reported that gardening activities lowered the odds ratio of depression/anxiety disorders. Many previous studies have reported the positive effects of gardening, such as how gardening may decrease depression/anxiety symptoms and improve mental health [27]. This study produced similar results.

Many previous studies focused on the beneficial effects of activities outside work. Particularly, research focused on the health effects of leisure time physical activity (LTPA) during leisure time. LTPA lowers the occurrence of obesity [28], mortality, and cardiovascular disease [29]. Additionally, LTPA during pregnancy has the effect of lowering the risk of preeclampsia and gestational hypertension [30]. Furthermore, steady LTPA lowers the risk of pancreatic cancer in young people [31], and certain studies show it can prevent various types of cancer [32]. Studies on mental disorders have also found that LTPA can lower the risk of postpartum depression [33], depressive symptoms [34], and help sleep initiation in elderly people [35]. However, no specific relationships were found in this study. The reason may be that in the KWCS, cultural activities were also included (along with sports and exercise) as an independent variable and so, the range of scope increased.

One strength of this study is that this was the first time that the relationship between activities outside work of workers with sleep disorders and depression/anxiety disorders was analyzed using the Republic of Korean data. There were many studies on activities outside work relating to mental health of workers in foreign countries. However, this study is the first research attempting to find out how activities outside work, which are major indicators of well-being, affect the mental health of workers in the Republic of Korea, and so the researchers of this study believe this to be important.

Second, the study was able to confirm that results from previous research conducted in foreign countries did not yield the same effects in South Korea. The health effects of activities outside work do not necessarily affect South Korean wage workers positively. Thus, this study was able to highlight the necessity of further research on this matter. The researchers of this study believe further research is needed to reflect the unique characteristics of the Republic of Korea.

This study, however, has limitations. First, as a cross-sectional study, it did not reveal the causality between leisure and social activities with sleep disorders and depression/anxiety disorders. Though it did present a relationship that did not previously exist, and so it was meaningful in the sense that new alternatives were presented.

Second, in the KWCS, general characteristics such as personal history, drinking, smoking, and other factors that may affect sleeping or depression/anxiety were excluded from the questionnaire items and were not included in the adjustment. However, the researchers of the study believe the value of this research to be sufficient as there are already many studies based on existing Working Conditions Surveys and European Work Environment Surveys.

Third, the definition of sleep disorders and depression/anxiety disorders may lack objectivity, as the survey was a self -report questionnaire. However, many of the existing studies have used self-report questionnaires to show sufficient, valid results.

Although there are some limitations as mentioned above, the research results will be a valuable resource towards discovering new alternatives for improving the mental health of Korean wageworkers.

## Conclusion

The study found how activities outside work such as volunteer activities, self-development activities, sports/cultural activities, and gardening activities in South Korean wageworkers are related to sleep disorders and depression/anxiety disorders. Contrary to previous research results, voluntary activities and self-development activities were shown to increase risk of sleep disorders or depression/anxiety disorders. The relationships with gardening activities were consistent with previous studies, reducing risk of depression/anxiety disorders. The results of this study suggest that the participation of activities outside work of the Republic of Korean wage workers negatively affect mental health. Therefore, a systematic review and preparation of countermeasures for the causes leading to these results are needed in subsequent studies.

## Abbreviations

CI: Confidence Interval
EWCS: European Working Conditions Survey
KOICD: Korea Informative Classification of Diseases
KRW: Korean Won
KWCS: Korean Working Conditions Survey
LTPA: Leisure time physical activity
OR: Odds Ratio
OSHRI: Institute Occupational Safety and Health Research

## References

[1] MJ C: The epidemiological survey of mental disorders in Korea. (Research IS ed. Policy Research Information Service & Management; 2011.

[2] Kim G, Min B, Jung J, Paek D, Cho S-i (2016). The association of relational and organizational job stress factors with sleep disorder. Ann Occup Environ Med.

[3] Grandner MA, Alfonso-Miller P, Fernandez-Mendoza J, Shetty S, Shenoy S, Combs D (2016). Sleep: important considerations for the prevention of cardiovascular disease. Curr Opin Cardiol.

[4] Lee IK. Economic Cost of Sleep Disorders. J Korean Sleep Res Soc. 2012;9:1–4.

[5] Chu C, Hom MA, Rogers ML, Stanley IH, Ringer-Moberg FB, Podlogar MC, Hirsch JK, Joiner TE (2017). Insomnia and suicide-related behaviors: A multi-study investigation of thwarted belongingness as a distinct explanatory factor. J Affect Disord.

[6] Laube I, Seeger R, Russi E, Bloch K (1998). Accidents related to sleepiness: review of medical causes and prevention with special reference to Switzerland. Schweizerische Medizinische Wochenschrift.

[7] Chilcott LA, Shapiro CM (1996). The socioeconomic impact of insomnia. PharmacoEconomics.

[8] Rutledge T, Reis VA, Linke SE, Greenberg BH, Mills PJ (2006). Depression in heart failure: a meta-analytic review of prevalence, intervention effects, and associations with clinical outcomes. J Am Coll Cardiol.

[9] Lett HS, Blumenthal JA, Babyak MA, Sherwood A, Strauman T, Robins C, Newman MF (2004). Depression as a risk factor for coronary artery disease: evidence, mechanisms, and treatment. Psychosom Med.

[10] Cizza G, Primma S, Csako G (2009). Depression as a risk factor for osteoporosis. Trends in Endocrinol Metab.

[11] Alder J, Fink N, Bitzer J, Hösli I, Holzgreve W (2007). Depression and anxiety during pregnancy: a risk factor for obstetric, fetal and neonatal outcome? A critical review of the literature. J Matern Fetal Neonatal Med.

[12] Pfeiffer PN, Ganoczy D, Ilgen M, Zivin K, Valenstein M (2009). Comorbid anxiety as a suicide risk factor among depressed veterans. Depression and anxiety.

[13] Reddy M (2010). Depression: the disorder and the burden. Indian J Psychol Med.

[14] Lee SH, Lee HS, Kim GH, Lee J-H, Lee K-J, Kim JJ (2016). The association between perceived discrimination and depression/anxiety disorders among Korean workers: analysis of the third Korean Working Conditions Survey. Ann Occup Environ Med.

[15] Kim H-C, Kim B-K, Min K-B, Min J-Y, Hwang S-H, Park S-G (2011). Association between job stress and insomnia in Korean workers. J Occup Health.

[16] Kim BH, Lee H-E (2015). The association between working hours and sleep disturbances according to occupation and gender. Chronobiol Int.

[17] Doljansky J, Kannety H, Dagan Y (2005). Working under daylight intensity lamp: an occupational risk for developing circadian rhythm sleep disorder?. Chronobiol Int.

[18] Sloman L, Gilbert P, Hasey G (2003). Evolved mechanisms in depression: the role and interaction of attachment and social rank in depression. J Affect Disord.

[19] Shah N, Eisner T, Farrell M, Raeder C. An overview of SSRIs for the treatment of depression. J Pharm Soc Wisc. 1999:33–49.

[20] Raphael B. Unmet need for prevention. Unmet Need in Psychiatry Probl, Resour, Responses. 2000:132–45.

[21] Sonnentag S (2001). Work, recovery activities, and individual well-being: a diary study. J Occup Health Psychol.

[22] Tucker P, Dahlgren A, Akerstedt T, Waterhouse J (2008). The impact of free-time activities on sleep, recovery and well-being. Appl Ergon.

[23] Nasermoaddeli A, Sekine M, Kumari M, Chandola T, Marmot M, Kagamimori S (2005). Association of sleep quality and free time leisure activities in Japanese and British civil servants. J Occup Health.

[24] Pavlova MK, Silbereisen RK (2012). Participation in voluntary organizations and volunteer work as a compensation for the absence of work or partnership? Evidence from two German samples of younger and older adults. J Gerontol Ser B Psychol Sci Soc Sci.

[25] McWilliams A, Siegel D (2001). Corporate social responsibility: A theory of the firm perspective. Acad Manag Rev.

[26] Han J, Koo J. Persistence or Change? Patterns of Work Schedules in Korea, 1990~ 2010. Korean. Soc Sci. 2013;47:69–82.

[27] Clatworthy J, Hinds J, Camic M. P: Gardening as a mental health intervention: a review. Ment Health Rev J. 2013;18:214–25.

[28] Sarma S, Zaric GS, Campbell MK, Gilliland J (2014). The effect of physical activity on adult obesity: Evidence from the Canadian NPHS panel. Econ Hum Biol.

[29] Cheung YK, Moon YP, Kulick ER, Sacco RL, Elkind MS, Willey JZ. Leisure-time physical activity and cardiovascular mortality in an elderly population in northern Manhattan: a prospective cohort study. J Gen Intern Med. 2016:1–7. 10.1007/s11606-016-3884-y PMC5264679 27752879

[30] Saftlas AF, Logsden-Sackett N, Wang W, Woolson R, Bracken MB (2004). Work, leisure-time physical activity, and risk of preeclampsia and gestational hypertension. Am J Epidemiol.

[31] Farris MS, McFadden AA, Friedenreich CM, Brenner DR: The association between leisure time physical activity and pancreatic cancer risk in adults: a systematic review and meta-analysis**.** Cancer Epidemiology and Prevention Biomarkers 2015**:**cebp 0301.2015. 10.1158/1055-9965.EPI-15-0301 26174790

[32] Amin TT, Al-Hammam AM, AlMulhim NA, Al-Hayan MI, Al-Mulhim MM, Al-Mosabeh MJ, Al-Subaie MA, Al-Hmmad QA, Al-Omran AA (2014). Physical activity and cancer prevention: awareness and meeting the recommendations among adult Saudis. Asian Pac J Cancer Prev.

[33] Teychenne M, York R (2013). Physical activity, sedentary behavior, and postnatal depressive symptoms: a review. Am J Prev Med.

[34] Chi SH, Wang JY, Tsai AC (2016). Combined association of leisure-time physical activity and fruit and vegetable consumption with depressive symptoms in older Taiwanese: Results of a national cohort study. Geriatr Gerontol Int.

[35] Kitano N, Tsunoda K, Tsuji T, Osuka Y, Jindo T, Tanaka K, Okura T (2014). Association between difficulty initiating sleep in older adults and the combination of leisure-time physical activity and consumption of milk and milk products: a cross-sectional study. BMC Geriatr.

